# Systematic characterization of cleanroom-free fabricated macrovalves, demonstrating pumps and mixers for automated fluid handling tuned for organ-on-chip applications

**DOI:** 10.1038/s41378-022-00378-y

**Published:** 2022-05-23

**Authors:** Elsbeth G. B. M. Bossink, Anke R. Vollertsen, Joshua T. Loessberg-Zahl, Andries D. van der Meer, Loes I. Segerink, Mathieu Odijk

**Affiliations:** 1 BIOS Lab on a Chip Group, MESA+Institute, Technical Medical Center, Max Planck Institute for Complex Fluid Dynamics, University of Twente, Enschede, The Netherlands; 2 Applied Stem Cell Technologies Group, Technical Medical Center, University of Twente, Enschede, The Netherlands

**Keywords:** Microfluidics, Microengraving

## Abstract

Integrated valves enable automated control in microfluidic systems, as they can be applied for mixing, pumping and compartmentalization purposes. Such automation would be highly valuable for applications in organ-on-chip (OoC) systems. However, OoC systems typically have channel dimensions in the range of hundreds of micrometers, which is an order of magnitude larger than those of typical microfluidic valves. The most-used fabrication process for integrated, normally open polydimethylsiloxane (PDMS) valves requires a reflow photoresist that limits the achievable channel height. In addition, the low stroke volumes of these valves make it challenging to achieve flow rates of microliters per minute, which are typically required in OoC systems. Herein, we present a mechanical ‘macrovalve’ fabricated by multilayer soft lithography using micromilled direct molds. We demonstrate that these valves can close off rounded channels of up to 700 µm high and 1000 µm wide. Furthermore, we used these macrovalves to create a peristaltic pump with a pumping rate of up to 48 µL/min and a mixing and metering device that can achieve the complete mixing of a volume of 6.4 µL within only 17 s. An initial cell culture experiment demonstrated that a device with integrated macrovalves is biocompatible and allows the cell culture of endothelial cells over multiple days under continuous perfusion and automated medium refreshment.

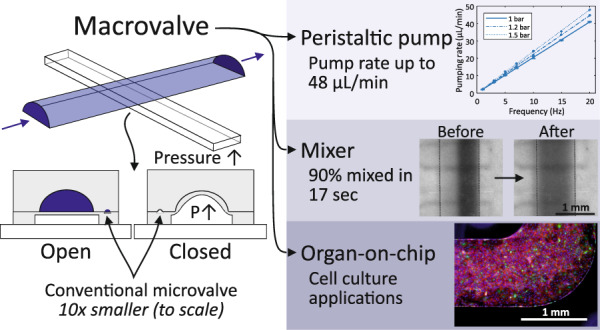

## Introduction

Organ-on-chips (OoCs) are commonly defined as microfluidic cell culture devices containing two independently addressable, parallel channels that are separated by a porous membrane. Different cell types can be cultured on both sides of the membrane, resulting in a complex, organ-specific, tissue-tissue interface^1,2^. OoC devices are considered to be a powerful alternative to conventional in vitro and animal models^3^. However, performing on-chip cell culture experiments is not trivial. OoCs can be labor intensive and challenging to use, as they require experience in both microfluidics and cell culturing^4,5^.

To translate OoCs from proof-of-concept devices into commercial systems, e.g., drug screening and personalized medicine, it is crucial that the OoC systems have a higher throughput. Multiplexed OoCs are a promising approach for increasing the throughput of OoC experiments^5,6^. Over the last few years, several microfluidic systems have been presented with a higher level of parallelization or throughput, but each of them has their own drawbacks. For instance, Mimetas OrganoPlate® is a system with 40 to 96 independent culture wells or OoCs^7^. However, it requires many pipetting steps to fill each individual chip, the cell culture area is small, and the setup requires the use of a hydrogel (as a semipermeable barrier and/or as a cell substrate). Zakharova et al. showed an example of a design with one common entrance and eight parallel outputs that can be used to achieve higher levels of throughput, but a lot of manual handling is still necessary^8^.

Systems with integrated microfluidic valves are often used to reduce the need for manual liquid handling, such as those shown by Vollertsen et al.^9,10^. These systems often use integrated normally open valves^11^, as the valves are both easy to fabricate and have small footprints relative to the channel widths when compared to those of normally closed valves^12,13^. In 2000, Unger et al. presented a normally open PDMS valve that is currently often used, also known as Quake-style valves^12^. These microvalves are an essential tool for automated control in microfluidics, as they can be applied for mixing, pumping, and multiplexing purposes in a broad range of applications^9,10,14,15^. Although these microfluidic large-scale integration (mLSI) systems enable a higher throughput, they are not compatible with the large channel dimensions that are needed to accommodate relevant cell cultures in OoCs.

The original fabrication process of Quake-style valves relies on a reflow photoresist to achieve rounded channels for the full sealing of the valves, limiting its application to channel heights up to tens of micrometers^12^. This makes the valves unsuitable for most OoC applications, as OoCs often contain channels hundreds of micrometers high and wide^16–20^. In addition, 3D cell culture systems, such as cell spheroids in a PDMS chip, often require channel dimensions of hundreds of micrometers^21^. Moreover, the peristaltic pumps that are fabricated using a reflow photoresist can usually only achieve flow rates from 0.05 µL/min up to 0.15 µL/min^12,14,22–24^. However, the flow rates typically used in OoCs are an order of magnitude higher, between 0.5 µL/min and 3.3 µL/min^16–20,25^, which can be even higher in blood vessels-on-chips to achieve physiologically relevant shear stresses. To transfer the advantages of mLSI chip technology into OoC technology, new fabrication methods for normally open PDMS valves are needed.

Although the use of PDMS is under debate^13,26,27^, it remains the material of choice for many research groups:^28^ it is easy to use in fabrication processes (for casting, surface coating, and bonding to PDMS or glass), gas permeable (allowing oxygen to diffuse to the cells), low cost, and it has a high elasticity (allowing the possibility for Quake-style valves)^13,26,29^. Previously, new fabrication methods using photolithography and 3D printing have been explored to create valves that can close off channels hundreds of micrometers high. Freitas et al.^30^ has shown a method to fabricate a Quake-style valve using photolithography with a maximal height and width of approximately 250 and 400 µm, respectively. However, their method requires extra soft lithography steps and pressurizing the channels during PDMS curing^30^, complicating the fabrication process. 3D printing also offers an alternative to photolithography for the fabrication of Quake-style valves, as shown by Lee et al.^31^ and Glick et al.^32^. However, these valves are fully 3D printed and not made from PDMS, which means that the valves cannot be directly integrated in a PDMS OoC^31^. Glick et al. and Compera et al. both show a Quake-style valve, closing a 500 µm high and 200 µm high channel, respectively, fabricated by 3D printed direct molds^32,33^. However, the 3D printing of molds for PDMS soft lithography poses challenges in terms of material compatibility, surface finish and the inability to vapor polish, resolution limit, repeatability, ease of use and fabrication speed^34^, and the use of a photocrosslinker, which can interfere with the curing of PDMS^35,36^.

Micromilling is a rapidly emerging rapid prototyping technique for fabricating microfluidic molds that offers a unique set of advantages over 3D printing and photolithography. Micromilling is a fast, versatile, cleanroom-free, and thus low-cost fabrication method, allowing complex 3D geometries within one mold^37^. A micromilled mold made of a plastic, such as polymethylmethacrylate (PMMA), is stronger and thus more resilient than fragile photoresist masters, which are particularly vulnerable when relatively large photoresist structures are obtained. Furthermore, micromilling of molds is less labor intensive than fabricating molds by the conventional process of photolithography using reflow photoresist, which also requires experience to achieve reproducible results. Although micromilled molds for PDMS macrovalves have already been reported^21,38^, the corresponding fabrication methods require a negative mold and double casting of PDMS on PDMS^21^, or a polyethylene terephthalate (PET) casting step^38^. In addition, the dimensions of these rounded channels are limited by the availability of cone-shaped milling tools^21,38^.

To our knowledge, we are the first to describe a method in which micromilling is used to directly fabricate a positive mold for a Quake-style PDMS macrovalve with dimensions in the hundreds of micrometers. Our proposed method allows us to create a mold with an upstanding structure with any shape (e.g., cylindrical or elliptical) and size (height and width) as desired, without being limited by the shapes and sizes of the ball or cone mills available. Such direct positive molds facilitate and simplify the fabrication process and minimize the potential error caused by PDMS shrinkage compared to the double casting methods^21,38^. In summary, the proposed micromilled direct molds for fabricating Quake-style valves allow an easier approach for the commercialization of molds and the multiplexing of PDMS OoCs.

In this article, we show a completely cleanroom-free fabrication method for Quake-style PDMS ‘macrovalves’ by using micromilling to fabricate direct positive molds. The control channels can form both bridges to cross as well as valves to close off rounded flow channels. In addition, we offer a valve design guide by systematically characterizing the bridge and valve channel heights, widths and actuation pressures. The macrovalves can close off rounded channels that are up to 700 µm high and 1000 µm wide. One macrovalve design (for closing a 400 µm high, 1000 µm wide rounded channel) was used for further experiments. By creating both a peristaltic pump and a mixing and metering device, we demonstrated the effectiveness of the macrovalve. The peristaltic pump can achieve a pumping rate of up to 48 µL/min, and the mixing and metering device can achieve the complete mixing of a volume of 6.4 µL within only 17 s. A second valve design (for closing a 200 µm high, 1000 µm wide channel) was used for a proof-of-concept cell culture experiment showing the culture of endothelial cells over multiple days under peristaltic flow in a PDMS device, demonstrating its biocompatibility. This experiment also demonstrates the potential of the macrovalves to be applied in multiplexed OoCs and allow the automation of cell culture in OoCs, which is extremely relevant for obtaining higher throughput OoC research and simultaneously reducing the need for manual liquid handling.

## Results

### Fabrication of PDMS devices with integrated macrovalves

**Fig. 1:**
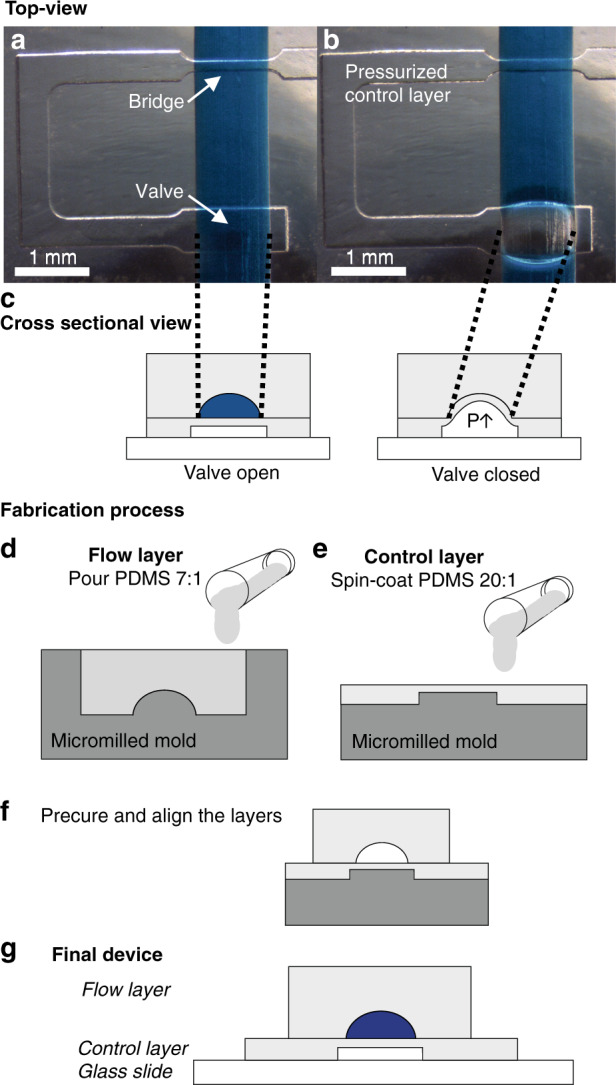
Fabricated PDMS macrovalve. **a**, **b** Top-view microscopic pictures of: **a** a control layer with an open valve and a bridge, **b** a pressurized control channel with a closed valve and a bridge, not closing the flow channel. The rounded flow channels contain blue food coloring, and the control channels contain water. **c** Schematic illustration of an open and a closed valve from a cross sectional view at the site of the valve. By pressurizing the control channel (*P* ↑ ), the valve closes. **d**–**g** Simplified illustration of the fabrication process. **d** A micromilled mold with an upstanding, rounded structure is used to cast PDMS as flow layer. **e** A micromilled mold with rectangular structures (control channels) is used as control layer. PDMS can be spin-coated on the mold, resulting in a thin PDMS membrane between the control channel and the flow channel. **f** PDMS can be precured, and the two layers can be subsequently aligned. **g** The control layer is bonded to a glass slide. The final fabricated device consists of three layers; a glass slide, the control layer, and the flow layer

The fabricated devices with integrated macrovalves (Fig. 1) all consisted of three layers (Fig. 1g): a glass slide, a thin PDMS layer containing control channels that were covered by a flexible PDMS membrane (control layer), and a PDMS layer with a rounded flow channel (flow layer). Figure 1a, b shows a control channel crossing a 1 mm wide, 400 µm high, rounded flow channel. The flow channel was filled with blue food dye. If the cross section of the control channel with the flow channel was sufficiently wide, the membrane of the control channel deflected upon pressurization into the flow channel and closed it off (Fig. 1b, c), thereby closing the valve. The (simplified) fabrication process is illustrated in Fig. 1d–g. Directly micromilled molds were used for casting the PDMS for the flow layer and spin coating the PDMS for the control layer. After precuring both layers, they can be aligned and bonded to a glass slide based on Unger et al.^12^.

### Reducing the surface roughness of the micromilled mold

**Fig. 2:**
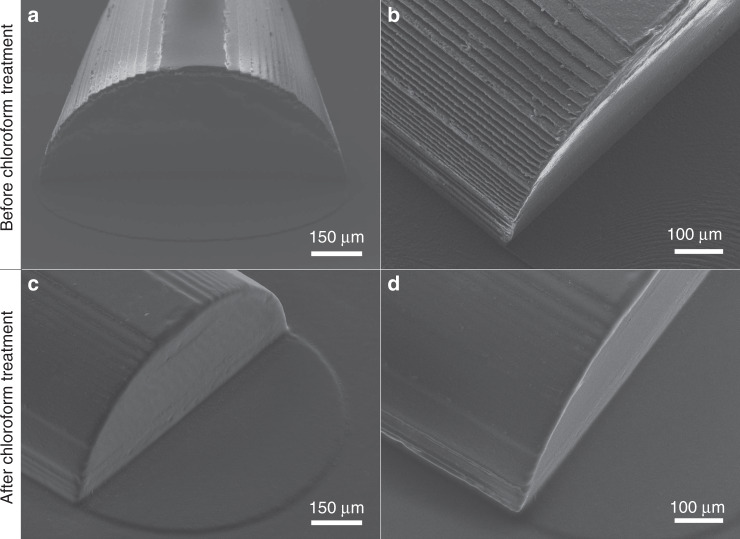
SEM images of the PMMA mold for the rounded flow channel: before (**a**, **b**) and after (**c**, **d**) chloroform solvent treatment

To obtain the full sealing of the valve, the flow channel should have a rounded, ideally smooth profile. Scanning electron microscopy (SEM) images were taken, showing a staircase-like structure in the PMMA mold (Fig. 2a, b) as a result of milling this protruding rounded structure. With a Dektak profilometer (Veeco), the surface profile of the milled, rounded structure in the PMMA mold was measured (Supplementary information S.2). The vertical steps of the staircase-like structure were measured to be 10 µm. A cross section of a PDMS cast of the flow channel is shown in Fig. S1. To reduce the surface roughness of the channel, a 5-minute chloroform treatment of the PMMA molds was performed based on Ogilvie et al.^39^. (Supplementary information S.1, Solution 1B). The SEM images (Fig. 2c, d) show the smoothing effect of the solvent treatment on the surface of the rounded protruding structure. This smoothing effect was also seen in a Dektak measurement of the treated mold (Supplementary information S.2, Fig. S3). We found that the valves were leak-free when closed (limit of detection: 10 nL/min) and usable for pumping regardless of the additional smoothing step. Hence, we did not use chloroform treatment for the systematic characterization chips, the peristaltic pump or the mixing and metering device. Smoothing is performed on the molds for the recirculation chip that is used for cell culturing.

**Fig. 3:**
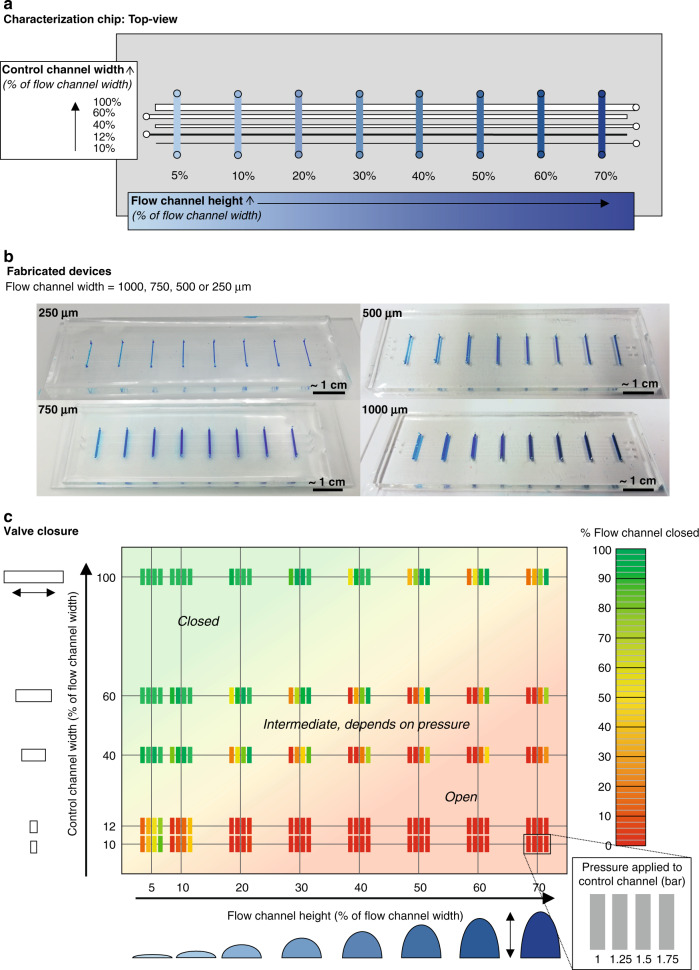
Systematic characterization of the valves. **a** Schematic top-view of the chip for characterization. This chip is fabricated in 4 versions, each with a fixed flow channel width (250, 500, 750, or 1000 µm). The flow channel height is varied as a % of the flow channel width, indicated by the blue gradient. The control channel height is fixed per chip version, and the control channel width is varied as a % of the flow channel width. **b** Fabricated 4 versions of the chip, with the fixed flow channel width indicated. **c** Valve closures of the 4 chip versions at different actuation pressures (indicated by the 4 vertical bars at the bottom right: 1, 1.25, 1.5, 1.75 bar) (per bar, *n* = 9 to 12). The valve closures for the separate chip versions (250, 500, 750, and 1000 µm flow channel width) can be found in Supplementary information S.5

### Systematic characterization of macrovalve actuation as a function of dimension and pressure

A systematic characterization of valves with different dimensions and/or control line pressures provides a useful design tool for manufacturing PDMS devices with various dimensions and applications. This systematic characterization determines the required control channel width for ranges of flow channel widths and heights at different actuation pressures. Four characterization chips (each containing 25 cross sections between a control channel and a flow channel) were designed and fabricated. For each chip, the width for the rounded flow channel was fixed (250, 500, 750, or 1000 µm wide; see Fig. 3a, b). A range of heights for the flow channels, which are given as a percentage (%) of the flow channel width, was examined. Furthermore, five control channels with a range of widths, also all given as a percentage (%) of the flow channel width, were examined. Cross sections of the PDMS casts of the micromilled molds can be found in Supplementary information S.3 and S.4 for the flow and control channels, respectively.

The flow channels were filled with blue food dye, and a range of actuation pressures (1, 1.25, 1.5, and 1.75 bar) was applied to the control channels. The control/flow channel cross sections were observed under a microscope for the presence of blue food dye in the flow channel (as shown in Fig. 1b). Figure 3c shows the percentage of valve closure averaged for all 4 chip versions at the different actuation pressures. Figure 3c shows that our proposed fabrication method worked to close off different sizes of flow channels, with heights of up to 70% of the width. With a narrow control channel (width of 10–12% of the flow channel width), bridging a flow channel was never a problem as long as the flow channel height was higher than 20% of the width. For closing, a control channel as wide as the flow channel seemed to be the most robust, although they had the disadvantage of a large footprint. When a small footprint is preferred, a control channel width of 60% of the flow channel width also sufficed at higher actuation pressures.

As OoCs often have a 1 mm wide channel with a height varying from 150 µm up to 1 mm^16–20,25^, we chose to further examine a valve fitting these OoC dimensions. In the remainder of this paper, we examined the valve closing of a 1000 µm wide and 400 µm high channel, henceforth referred to as the ‘macrovalve’.

### Macrovalve actuation and leakage

**Fig. 4:**
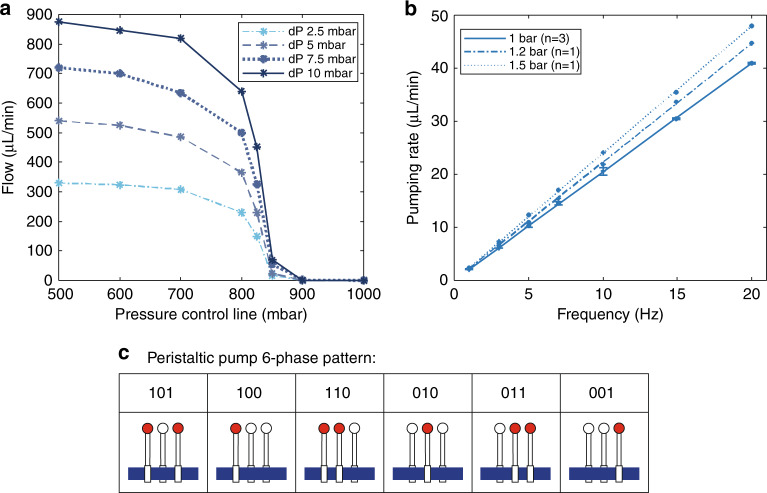
Characterization of a peristaltic pump. **a** Closing behavior of the macrovalve. Lines for visual guidance. dP = differential pressure between inlet and outlet pressures of the flow channel, which are variated and indicated by the different colors (*n* = 1). **b** Pumping rate of the peristaltic pump at different frequencies and pressures of actuation with a 6-phase pattern (101, 100, 110, 010, 011, 001). A linear relation is observed between the pumping rate and the actuation frequencies for all three actuation pressures. 1 bar, *R*^2^ = 0.9999 (*n* = 3); 1.2 bar, R^2^ = 0.9997 (*n* = 1); 1.5 bar, *R*^2^ = 0.9998 (*n* = 1). **c** Schematic illustration of the 6-phase actuation pattern of the peristaltic pump

The closing behavior of the macrovalve was examined by applying different pressures to the flow and control channels. Two pressure regulators were used to apply a differential pressure (dP) to the inlet and outlet of the flow channel, and one pressure regulator was used to apply pressure to the control channel. A flow sensor, in series with the valve, was used to measure the flow at different pressures (see Fig. 4a). By applying more than 900 mbar to the control line, the valve could be closed for all four input pressures ranging from 2.5 mbar to 10 mbar.

Valve leakage was also examined and is shown in Supplementary information S.6. Simply put, the average valve leakage is in the range of nL/min, which is only 0.1% of the flow rate with an open valve, from which we could conclude that the leakage of a closed valve is negligible for our applications.

### Peristaltic pumping rate

Three consecutive valves were used to create a peristaltic pump. The pumping rate of the peristaltic pump was examined by the actuation of the valves with a 6-phase pattern (101, 100, 110, 010, 011, 001; schematically illustrated in Fig. 4c) at different frequencies. Figure 4b shows the linear response between the actuation frequency and the pumping rate for each actuation pressure. At a frequency of 20 Hz, a maximum pumping rate of 47.9 µL/min was achieved (see Fig. 4b). The measurements within one pump were reproducible, as indicated by the small, almost invisible error bars for the measurements at 1 bar. Another peristaltic pump, shown in Fig. S2, also showed a linear response. Frequencies above 20 Hz were not examined, as they cannot be achieved with the valve manifold setup used, which had a maximal switching frequency of 20 Hz.

### Mixing and metering device: mixing efficiency and dilution series

**Fig. 5:**
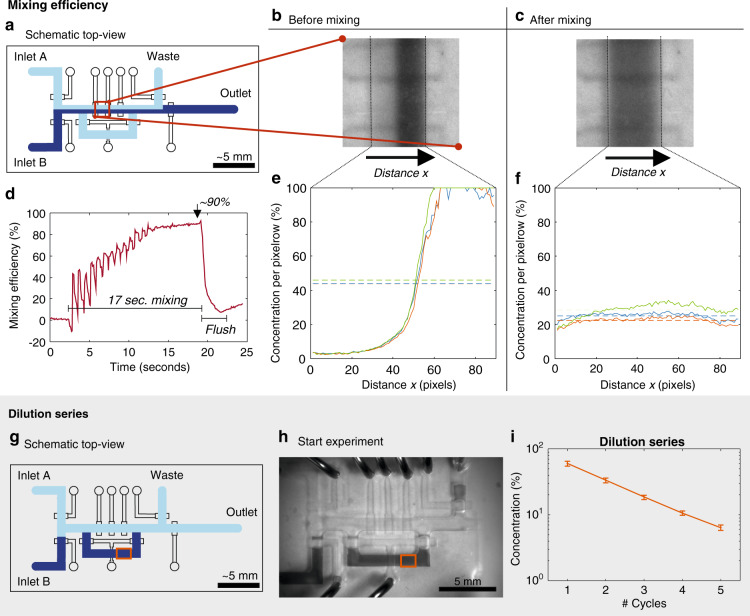
Mixing efficiency of a food dye (Inlet B) with water (Inlet A). **a** Schematic top-view of the device with an indication of the site where the intensity was measured. **b** Image of the channel before mixing. Channel width (indicated with the dashed lines) is 1 mm. **c** Image of the channel after mixing. Please note that this channel is rounded. Therefore, the intensity at the corners is lower, while the actual concentration is constant; see Supplementary information S.7 for the corresponding calibration.**d** Mixing efficiency over time. The periods of mixing and afterward flushing are indicated. After 17 s of mixing, the mixing efficiency approaches 90%. **e**, **f** Calculated concentration along the width of the channel (distance x) before (**e**) and after (**f**) mixing. An average mixing efficiency of 90.4 ± 3.32% was observed after 17 s of mixing (*n* = 3). Dilution of 100% food dye (Inlet B) with water (Inlet A). **g** Schematic top-view of the device with an indication (red box) of the site where the intensity was measured. **h** Image of the mixing and metering device at the start of the experiment. **i** Calculated concentration of the food dye in the channel after diluting 100% food dye with water for 5 cycles. An exponential decrease in food dye concentration is observed with an exponential fit of y = 102.69e^−0.561x^ with *R*^2^ = 0.999 (*n* = 3 measurements)

The mixing and metering device, schematically shown in Fig. 5a, contained two inlets, two outlets and an on-chip peristaltic pump. The mixing and metering device was characterized by mixing food dye and water in the flow channels of the chip. During mixing, images of the fluids in the chip were captured, which were later converted to concentrations using calibration curves, as further explained in the Materials and Methods section.

The amount of mixing can be quantified as the difference between the concentration profiles after mixing and the perfectly mixed case. When this is taken as a fraction of the difference between a completely unmixed profile (a step function) and the same perfectly mixed case, we arrive at the previously reported formulation of the mixing efficiency:^40^

1 $$Mixing\,efficiency = \left( {1 - \frac{{\sqrt {\frac{1}{N}{\sum} {\left( {c_{measured} - \bar c} \right)} ^2} }}{{\overline {\sqrt {\frac{1}{N}{\sum} {\left( {c_0 - \bar c} \right)} ^2} } }}} \right) \cdot 100\,{{{\mathrm{\% }}}},$$

where N is the number of pixel rows, *c*_*measured*_ is the concentration measured along the channel, $\bar c$ is the average concentration along the channel and *c*_0_ is the completely unmixed concentration profile along the channel. For this *c*_0_ profile, we take a step function from 0 for half the channel width to 2$\bar c$ for the other half of the channel width as a reference for the unmixed state, as described by Johnson et al.^40^.

Figure 5a schematically shows the site where the intensity was measured and the concentration was calculated. Figure 5b, c shows the actual pictures before and after mixing, with the calculated concentration along the distance x shown in Fig. 5e, f. The mixing efficiency improved from 3.46 ± 1.61% before mixing to 90.4 ± 3.32% after 17 s of mixing. The mixing efficiency was also calculated over time, as shown in Fig. 5d. A video recording of the mixing process can be found in Supplementary information S.8.

The mixing and metering device was also used to perform a dilution series (Fig. 5g–i). First, the flow channels of the device shown in Fig. 5g were filled with food dye, and after a valve closed the mixing loop, the main channel was subsequently flushed with 100% water (Fig. 5g, h). This water was mixed for 17 s with the food dye present in the mixing loop, diluting the food dye, which was repeated 5 times, indicated as dilution cycles. A video recording of the dilution series performance can be found in Supplementary information S.9. The concentration in the channel was calculated with the same method as that for the mixing experiments (further explained in the Materials and Methods and Supplementary information S.7). The concentration in the channel is plotted per cycle of dilution in Fig. 5i. The dilution series shows an exponential fit (*R*^2^ = 0.999), as expected, since for every cycle, approximately 37% of the total loop volume is replaced.

### On-chip endothelial cell culture under peristaltic flow

**Fig. 6:**
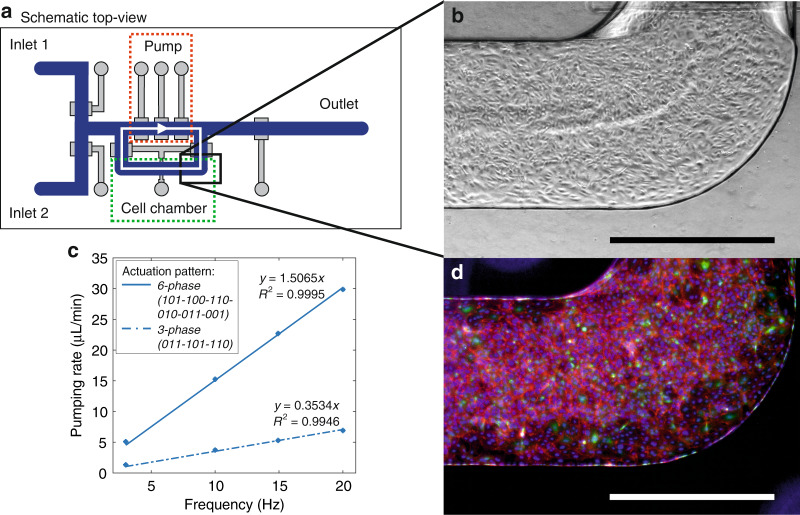
Endothelial cell culture in the recirculation chip^43^. **a** Schematic top-view of the device with indications of the peristaltic pump (red) and the cell chamber (green). The white arrows indicate the medium recirculation loop. **b** Phase contrast microscopic image of HUVECs (passage number 7) cultured on-chip for 96 h under constant peristaltic flow. Scale bar represents 1 mm. **c** Measured pumping rate of the on-chip peristaltic pump at different frequencies with 3-phase and 6-phase actuation patterns (for both, *n* = 1). **d** Fluorescence image of the GFP-expressing (green) HUVECs with stained cell nuclei (NucBlue) and F-actin (red). Scale bar represents 1 mm^43^

Both shear stress and constant exposure to paracrine signaling factors play important roles in the integrity of in vitro cultured endothelium. These two factors can be achieved by the recirculation of the cell culture medium in a chip either via an off-chip pump^41^ or an on-chip pump composed of microvalves^24,42^. The presented mixing and metering device allows recirculation of the medium in a closed loop. The ‘recirculation chip’ has a similar design to that of the mixing and metering chip (Fig. 6a), but the valves are 1 mm wide and the flow channel is 1 mm wide and 200 µm high^43^. The recirculation chip is used for a proof-of-concept experiment to culture endothelial cells for 96 h under peristaltic flow, applying a shear stress to the cells. During these 96 h, the macrovalves in the peristaltic pump were switched ON/OFF over 3 × 10^6^ times in total. The peristaltic pump was programmed to run at 10 Hz with a 3-phase (011-101-110) actuation pattern, resulting in a pumping rate of 3.7 µL/min (Fig. 6c, dotted line). This pattern was used for the initial cell culturing experiments to ensure a consistent volume of medium recirculating in the loop during pumping, as there are the same number of closed valves in each step of the pumping pattern. The same chip was also able to achieve higher pumping rates with the 6-phase actuation pattern (Fig. 6c, continuous line). The endothelial cells formed a confluent cell layer in the flow channel 96 h after seeding (Fig. 6b, d).

## Discussion

### Fabrication process

Our proposed fabrication method (briefly illustrated in Fig. 1d–g and described in detail in the Materials and Methods section) provides an approach for the fabrication of a macrovalve using two positive molds with only one soft lithography step. This method completely relies on micromilling, which provides several advantages over the conventional photolithography process with a reflow photoresist. Micromilling gives the designer a large amount of freedom, as it allows for different heights within one mold, which would be much more cumbersome for photolithography masters, as this would require extra masks and fabrication steps. Furthermore, our proposed method is completely cleanroom-free, and the micromilled molds do not require priming or coating after fabrication. The time necessary for micromilling the two molds is dependent on the size of the mold and the amount and complexity of the structures in the mold. For the peristaltic pump and mixing and metering device demonstrated herein, the milling of both molds for one device took 1.5 h and 2 h, respectively. This is 3 times faster than the fabrication process of the photolithography technique with a reflow photoresist, which requires at least 6 h to fabricate both wafers.

The larger dimensions of our macrovalve allow a larger margin of error for the alignment of the two layers than those of typical microfluidic valves. The alignment can be performed by using a simple stereo microscope, or even by the naked eye, and it is not required for the user to be extremely precise or experienced. The dimensions (height and width) of the channels and valves are all one order of magnitude (10x) higher than those commonly obtained with the conventional reflow photoresist method.

### Reducing the surface roughness of the PMMA micromilled mold

The vertical ‘staircase’ steps of the rounded PMMA structure (Fig. 2a, b) were measured to be 10 µm, which can be tuned by a specific parameter in the HSMworks software (further explained in Supplementary information S.1, Solution 1A). A higher precision should be possible but requires more computational power and thus more computation (and milling) time. Another approach for smoothing the rounded structure is a chloroform solvent treatment based on Ogilvie et al.^39^ (Supplementary information S.1, Solution 1B). Figure 2 shows a clear smoothing of the PMMA mold surface due to this chloroform solvent treatment. However, the effect of chloroform solvent treatment can depend on several factors, such as the volume of the petri dish used, the volume and concentration of chloroform used and the distance between the chloroform liquid level and the surface of the PMMA mold. This results in the solvent treatment requiring optimization before it can be used, complicating the fabrication process. We found that both additional smoothing options were not required to close off the flow channel completely.

### Systematic characterization of macrovalve actuation as a function of dimension and pressure

The systematic characterization performed (Fig. 3 and Supplementary information S.3–S.5) provides a valuable design tool for fabricating valves with various sizes and/or applications. Please note that this valve closure is highly dependent on the thickness of the thin PDMS membrane between the control and flow layers. When a different spinning speed, PDMS ratio, size mold, or PDMS viscosity is used, this thickness can be different, leading to differences in valve characteristics (see Supplementary information S.1 Problem 2–4). We recommend using the proposed fabrication technique for closing off rounded channels with widths of 500 µm or larger, as both the control layer and the flow layer of the 250 µm chip have very high percentage deviations in both their widths and heights (Supplementary information S.3–S.5).

### Macrovalve actuation and leakage

The valve leakage when applying a 20 mbar pressure to the flow channel and a 1.5 bar pressure to the control channel is in the range of nL/min, which is only 0.1% of the flow rate with an open valve and below the limit of detection (10 nL/min) of the flow sensor used. The 90% response time from an open valve to a 90% closed valve was less than 0.5 s (Fig. S6). After these 0.5 s, the flow sensor is not sensitive enough to accurately measure. However, the flow is measured outside the PDMS chip with a macrovalve, which may cause a delay in the response time due to the inertia of the fluid. For compartmentalization purposes in OoC applications, this response time and valve sealing are sufficient.

### Peristaltic pumping rate

Figure 4b shows that the pumping rate increases as the actuation frequency increases, up until a frequency of 20 Hz, which is the maximum switching frequency of the valve manifold controlling the valves of the peristaltic pump^44^. The most commonly used flow rate for OoCs is 0.5 µL/min^18,19,25^ or 1 µL/min^16,17^, which can easily be obtained by the presented peristaltic pump. We report a maximum pumping rate of 48 µL/min, which is much higher than the pumping rates reported for peristaltic pumps fabricated by the conventional reflow photoresist method. The pumping rates usually achieved by these pumps range from 0.05 µL/min up to 0.15 µL/min^12,14,22,23^. Pumping rates of 7.5 µL/min for devices using conventional Quake-style valves are documented^45^. However, these pumps require very high frequencies (300–400 Hz) to obtain these pumping rates, which cannot be obtained by the external solenoid valve manifold used in this research. High-speed electrovalves, as used by Goulpeau et al., could resolve this problem. However, the attainable pressure switch time is also limited by the actuation volumes (i.e., the volumes of the tubing and the control channel)^45^.

Figure 4b shows the almost perfectly linear response of the actuation frequency and its resulting pumping rate. Separate pumps show slightly different pumping rates at different frequencies and actuation pressures, but the aforementioned linearity facilitates the calibration. Depending on the desired flow rate, the peristaltic pump can be calibrated by determining a suitable actuation pressure and frequency, described in Supplementary information S.1, Solution 4 (Fig. S2). Within one pump chip, the flow-to-frequency plot is shown to be very reproducible (Figs. 4b, 1 bar).

### Mixing and metering device: mixing efficiency and dilution series

We showed that we could improve the mixing efficiency from 3.46 ± 1.61% before mixing to 90.4 ± 3.32% after 17 s of mixing (Fig. 5). In comparison, Kondapalli et al. showed a mixing and metering device with the application of refolding of a protein on-chip^24^, where they reported a required mixing time of 45 s to completely mix. However, the mixing efficiency has not been quantitatively reported.

A mixing efficiency above 90% is considered to indicate a uniform distribution along the channel^46^. However, in the literature, microfluidic mixers are passive mixers with a specific channel structure causing mixing and thus have a single mixing efficiency for a given flow rate. The mixing efficiency in our system is difficult to compare to those of the passive mixers in the literature for two reasons: first, mixing is actively achieved via recirculation, and second, the mixing loop contains a dead volume that is also recirculated during mixing. Both effects cause a change in mixing efficiency over time. The first effect means we can simply recirculate indefinitely to achieve increasingly better efficiencies. The second effect means that there are initially some oscillations in the measured mixing efficiency as the dead volume recirculates. To overcome this and still be able to facilitate a comparison to the literature, we instead characterized the time required for the oscillations to die out for the mixing efficiency to reach 90%. In Fig. 5d, we see that the oscillations die out after 10 s of recirculation, and a mixing efficiency of ~90% is reached after 17 s.

The dilution series shown in Fig. 5g–i shows an exponential fit, which is expected, since the dye was diluted with the same amount of water in each cycle. The fit shows a dilution factor of 0.561, which approaches the expected dilution factor of 0.584 obtained by calculating the volumes of the channels in the mixing loop (Supplementary information S.10). Other dilution factors can be achieved by adjusting the volume ratio in the chip.

### Endothelial cell culture under peristaltic flow

With the proof-of-concept cell culture experiment, we showed that the device is biocompatible and allows the cell culture of endothelial cells over multiple days. During the initial long-term experiments, we discovered that the bond between the control layer and the glass slide was not sufficiently strong to withstand actuation for more than 24 h. To solve this, we added an additional PDMS layer under the control layer, which is further described in the Materials and Methods section. In our initial cell culture experiment, we demonstrated that it is possible to actuate the valves over 96 h, during which we switched the valves ON/OFF over 3 × 10^6^ times.

The peristaltic pump generated a shear stress of approximately 0.01 Pa when actuated at 10 Hz according to a 3-phase pattern (calculated using an approximation of wall shear stress in rectangular channels^47^). This is not yet a physiologically relevant shear stress for blood vessels (>~0.5 Pa)^48–50^; however, this can be solved by reducing the cell chamber dimensions or by using the 6-phase pattern that was shown to achieve much higher pumping rates. For other organ-on-chip applications, such as gut-on-chips, the generated shear stress is already sufficient^25^.

### Conclusion and outlook

The presented cleanroom-free fabrication process based on the micromilling of a direct positive mold for Quake-style PDMS macrovalves is a method that, to our knowledge, has not been described before. We show that we can form both bridges to cross and valves to close off rounded channels of up to 700 µm high and 1000 µm wide. A systematic characterization of the valve and bridge dimensions is performed, which is a valuable design tool for devices with dimensions on the order of hundreds of micrometers (250–1000 µm) that cannot be achieved with the conventional reflow photoresist method typically used to produce Quake-style valves. The dimensions are specifically tuned for OoCs, and the results of an initial cell culture experiment support the conclusion that cells can be cultured with automated medium refreshment for at least multiple days by using this valve technology. In addition, the large stroke volumes of the macrovalves enable us to achieve pumping rates up to 48 µL/min using peristaltic pumping. The integration of these macrovalves will allow the multiplexing and automated control of cell culture conditions in OoCs. These parameters are essential for obtaining higher throughput OoCs while reducing the need for manual handling.

## Materials and methods

### PMMA mold fabrication

For each design, two PMMA molds were designed in 3D-CAD software (SolidWorks®, 2018) for the control and flow layers. The dimensions of the protruding structures depended on the chip design. The inlets and outlets were on a grid corresponding to the ISO Workshop Agreement 23:2016 standards^51^.

HSMworks, integrated CAD/CAM software in SolidWorks®, was used to program the milling steps. Specifically, the tolerance and smoothing settings in the design for the flow layer were essential for obtaining a smooth, rounded protruding structure (see Supplementary information S.1, Solution 1A). The PMMA stock material was micromilled (Datron Neo, Germany) to obtain the positive molds. For the flow layer, a 1 mm diameter mill was used, and for the smallest features in the control layer, a 0.4 mm diameter mill was used. An optional 5-minute chloroform solvent treatment of the PMMA molds based on Oglivie et al.^39^ can be performed (Supplementary information S.1, Solution 1B). After micromilling, dust can be removed by rinsing the molds with water (the use of an ultrasonic bath to remove eventual burrs and dust is optionally) and drying them using a nitrogen gun. The PMMA molds do not need priming or coating and can be directly used for PDMS casting.

### PDMS chip fabrication

**Fig. 7:**
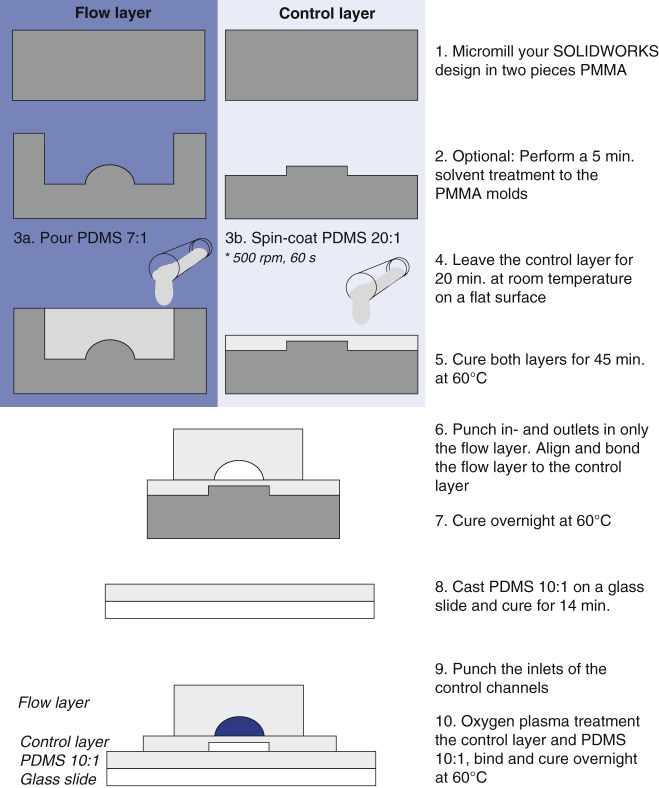
Schematic illustration of the fabrication processes of the PDMS devices. *Please note that the spinning speed in step 3b is dependent on the size of the PMMA control layer mold

The fabrication process for the PDMS devices, illustrated in Fig. 7, is based on Unger et al.^12^. PDMS (RTV 615, Permacol BV, the Netherlands) was mixed (1:7 (w/w)) for the flow layer and (1:20 (w/w)) for the control layer and subsequently degassed for 1.5 h. PDMS (1:7 (w/w)) was cast on the mold for the flow layer. For the control layer, PDMS (1:20 (w/w)) was spin-coated onto the micromilled PMMA mold for 60 s, resulting in an approximately 60 µm thick membrane at the valve site. The spinning speed required for this 60 µm thick membrane depended on the size of the mold and the control layer height. The spinning speeds used for the different chips are summarized, together with other relevant parameters for the fabrication protocol, in Supplementary information S.11 (Table S6). The control layer was placed on a flat surface after spinning for 20 minutes at room temperature, after which both layers were precured for 45 minutes at 60 °C. After precuring, inlets for the flow channel were punched with a 1 mm biopsy punch (Ted Pella, Inc., USA). Afterward, the two layers were aligned and pressed together to bond and cure overnight at 60 °C. Then, inlets for the control channels were punched with a 0.75 mm biopsy punch (Harris Uni-core), and the control layer was plasma-bonded to a glass slide. The final devices consisted of three layers: two PDMS layers (flow and control) and one glass layer.

While using the valves for cell culture over days, delamination of the control layer from the glass slide was observed. To solve this problem for the recirculation chip, an additional PDMS layer was added to the glass slide. PDMS was mixed (1:10 (w/w), Sylgard 184 Silicone elastomer kit, Dow corning), degassed and spin-coated on a glass slide at 300 rpm. This layer was precured for 14 minutes at 60 °C, after which the control layer and the PDMS-coated glass slide were oxygen plasma-treated. After bonding, the chip was fully cured at 60 °C. Preliminary results using an alternative method, as described in Supplementary information S.1, Problem 5, showed promising improvement for reliability and stability of the devices. This fabrication method will be explored in more depth in the future.

### Setup measurements

**Fig. 8:**
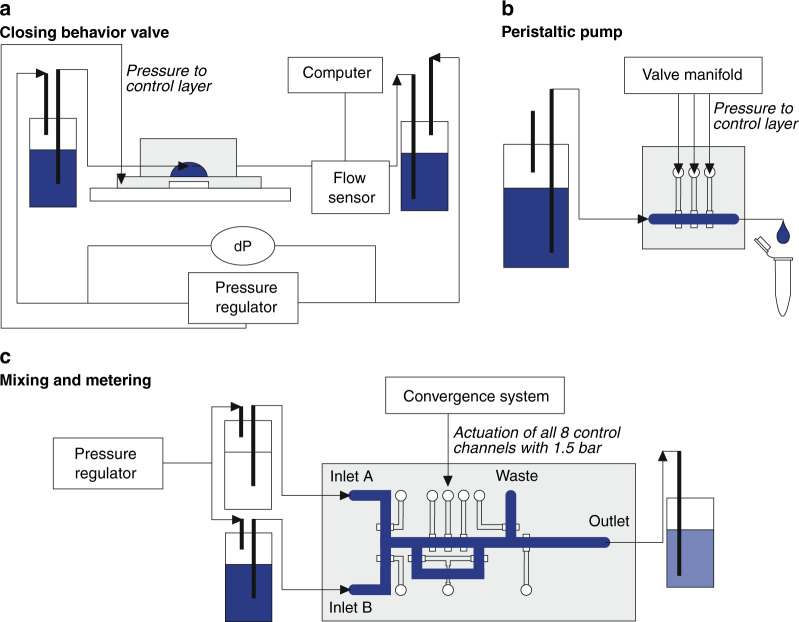
Schematic illustration of the experimental setups. Experimental setups for **a** testing the closing behavior of the valve, **b** measuring the pumping rate generated by the peristaltic pump and **c** measuring the mixing efficiency and dilution series

Figure 8a shows the experimental setup for testing the closing behavior of the valve. Two pressure regulators (Fluigent, LINEUP™ series) were used to apply pressures to the inlet and outlet of the flow channel, and the difference in these pressures is the differential pressure (dP). One pressure regulator (Fluigent, LINEUP™ series) was used to apply pressure to the control channel. In series with the valve, a flow sensor (Fluigent, FRP size L) was placed.

For the systematic characterization, the flow channels were filled with a blue food dye (JO-LA), and the valve closure was observed by using a microscope with a color CCD camera (FLIR Grasshopper 3, U23S6C) to record the images. A custom-made system (Convergence Industry B.V., the Netherlands) was used for the actuation of the control channels.

Figure 8b shows the experimental setup for measuring the peristaltic pumping rate. For the actuation of the three valves, the pressure to the control channels was applied via a valve manifold (Festo), which has a maximum switching frequency of 20 Hz and is controlled by an Easyport module (Festo). The peristaltic pump was actuated at a certain frequency and pressure for 5 or 10 minutes. The outflow was collected in Eppendorf tubes, which were weighed (Balance SX64, Mettler Toledo) before and after pumping.

For the mixing and metering device (Fig. 8c), a custom-made system (Convergence) was used for the actuation of the control channels. The control channels were actuated with a pressure of 1.5 bar. For mixing, the three valves were also actuated with a 6-pattern for 20 cycles at a frequency of 10 Hz. With a pressure regulator (Fluigent, LINEUP™ series), pressure was applied to both inlets of the mixing and metering device. Images were taken using a greyscale camera (Grasshopper3 GS3-U3-23S6M, Point Grey camera).

### Image processing

Image processing and analysis were performed using MATLAB (2017b). First, a background correction was performed to correct for overall intensity variations (Supplementary information S.7). A calibration session was conducted in which the flow channels of the mixing and metering device were filled with known concentrations of food dye diluted in water to obtain a calibration curve (Fig. S7). Fig. S7a shows that when the flow channel is filled with food dye, the measured intensity differs along the width of the channel (distance x in Fig. 5b, c) due to the rounded profile of the flow channel. To solve this, a calibration curve was established per pixel row. Three calibration curves at various sites along the width of the channel are shown in Fig. S7b–d, where (I_0_/I) is plotted against concentration. All calibration curves followed a second-order polynomial fit. Using these calibration curves, the unknown concentration along the channel could be calculated (Fig. 5). By analyzing the separate picture frames of the mixing process, the mixing efficiency was calculated over time (Fig. 5d). The average concentration ($\bar c$) was calculated per picture frame, which was used to calculate the mixing efficiency at that specific frame/time point.

### Endothelial cell culture

Green fluorescent protein (GFP)-expressing human umbilical vein endothelial cells (HUVECs) (Angio-Proteomie, USA) were cultured in endothelial growth medium (EGM) (Cell Applications, Inc., CA, USA) in a collagen I-coated T75 flask (CELLCOAT®, Greinder Bio-One). Prior to cell culture, the recirculation chip was sterilized by performing oxygen plasma-treatment (40 s, 50 Watt, Femto Science, Cute), and the flow channels were flushed with 70% ethanol (Boom, the Netherlands) and subsequently with phosphate-buffered saline (PBS, Sigma–Aldrich). GFP-expressing HUVECs (passage number 7) were seeded in the cell chamber at a seeding density of 4 × 10^6^ cells/mL. The on-chip peristaltic pump was programmed to run a 3-phase (011-101-110) actuation pattern at 10 Hz. The cell culture medium in the loop was partially replaced every 2 h automatically. The valves at inlet 1 and the outlet were opened, and the cell chamber closed (Fig. 6a), while the on-chip pump pumped fresh medium in the chip for 1 minute.

The on-chip culture experiment was carried out by placing the device in a custom-built incubation system^9^ for 96 h.

For fluorescence microscopy analysis, the HUVECs were fixed with 4% paraformaldehyde (Sigma Aldrich) in PBS and subsequently permeabilized with 0.3% Triton-X (Sigma Aldrich) in PBS. The cells were stained with 15 µL/mL of both AcinRed (Thermo Fisher Scientific) and NucBlue (Thermo Fisher Scientific) in PBS to visualize the F-actin filaments and the cell nuclei, respectively. Fluorescent and phase contrast images were captured using an EVOS FL cell imaging system.

## Supplementary information

Supplementary information

## References

[1] Ingber DE (2016). Reverse Engineering Human Pathophysiology with Organs-on-Chips. Cell.

[2] Bhatia SN, Ingber DE (2014). Microfluidic organs-on-chips. Nat. Biotechnol..

[3] Reardon S (2015). ‘Organs-on-chips’ go mainstream. Nature.

[4] Sontheimer-Phelps A, Hassell BA, Ingber DE (2019). Modelling cancer in microfluidic human organs-on-chips. Nat. Rev. Cancer.

[5] Probst C, Schneider S, Loskill P (2018). High-throughput organ-on-a-chip systems: Current status and remaining challenges. Curr. Opin. Biomed. Eng..

[6] Rothbauer M, Rosser JM, Zirath H, Ertl P (2019). Tomorrow today: organ-on-a-chip advances towards clinically relevant pharmaceutical and medical in vitro models. Curr. Opin. Biotechnol..

[7] Mimetas. Mimetas OrganoPlate. https://mimetas.com/page/products.

[8] Zakharova M (2020). Multiplexed blood-brain barrier organ-on-chip. Lab Chip.

[9] Vollertsen, A. R. et al. Modular operation of microfluidic chips for highly parallelized cell culture and liquid dosing via a fluidic circuit board. *Microsystems Nanoeng*. **6**, 107 (2020). 10.1038/s41378-020-00216-z PMC8433198 34567716

[10] Vollertsen, A. R. et al. Highly parallelized human embryonic stem cell differentiation to cardiac mesoderm in nanoliter chambers on a microfluidic chip. *Biomed. Microdevices* **23**, 30 (2021). 10.1007/s10544-021-00556-1 PMC8166733 34059973

[11] Thorsen T, Maerkl SJ, Quake SR (2002). Microfluidic Large-Scale. Integration.

[12] Unger MA, Chou HP, Thorsen T, Scherer A, Quake SR (2000). Monolithic microfabricated valves and pumps by multilayer soft lithography. Science.

[13] Ren K, Zhou J, Wu H (2013). Materials for microfluidic chip fabrication. Acc. Chem. Res..

[14] Gómez-Sjöberg R, Leyrat AA, Pirone DM, Chen CS, Quake SR (2007). Versatile, fully automated, microfluidic cell culture system. Anal. Chem..

[15] Blazek M, Santisteban TS, Zengerle R, Meier M (2015). Analysis of fast protein phosphorylation kinetics in single cells on a microfluidic chip. Lab Chip.

[16] Jalili-Firoozinezhad S (2019). A complex human gut microbiome cultured in an anaerobic intestine-on-a-chip. Nat. Biomed. Eng..

[17] Maoz BM (2017). Organs-on-Chips with combined multi-electrode array and transepithelial electrical resistance measurement capabilities. Lab Chip.

[18] Grassart A (2019). Bioengineered Human Organ-on-Chip Reveals Intestinal Microenvironment and Mechanical Forces Impacting Shigella Infection. Cell Host Microbe.

[19] van der Helm MW (2019). Non-invasive sensing of transepithelial barrier function and tissue differentiation in organs-on-chips using impedance spectroscopy. Lab Chip.

[20] Zhang YS (2017). Multisensor-integrated organs-on-chips platform for automated and continual in situ monitoring of organoid behaviors. Proc. Natl Acad. Sci. U. S. A..

[21] Silva Santisteban T, Rabajania O, Kalinina I, Robinson S, Meier M (2017). Rapid spheroid clearing on a microfluidic chip. Lab Chip.

[22] Bowen AL, Martin RS (2010). Integration of on-chip peristaltic pumps and injection valves with microchip electrophoresis and electrochemical detection. Electrophoresis.

[23] Cole MC, Desai AV, Kenis PJA (2011). Two-layer multiplexed peristaltic pumps for high-density integrated microfluidics. *Sensors Actuators*. B Chem..

[24] Kondapalli S, Kirby BJ (2009). Refolding of β-galactosidase: Microfluidic device for reagent metering and mixing and quantification of refolding yield. Microfluid. Nanofluidics.

[25] Kim HJ, Huh D, Hamilton G, Ingber DE (2012). Human gut-on-a-chip inhabited by microbial flora that experiences intestinal peristalsis-like motions and flow. Lab Chip.

[26] Campbell S (2020). Beyond polydimethylsiloxane: Alternative materials for fabrication of organ on a chip devices and microphysiological systems. ACS Biomater. Sci. Eng.

[27] van Meer BJ (2017). Small molecule absorption by PDMS in the context of drug response bioassays. Biochem. Biophys. Res. Commun..

[28] Volpatti LR, Yetisen AK (2014). Commercialization of microfluidic devices. Trends Biotechnol..

[29] Halldorsson S, Lucumi E, Gómez-Sjöberg R, Fleming RMT (2015). Advantages and challenges of microfluidic cell culture in polydimethylsiloxane devices. Biosens. Bioelectron..

[30] Freitas, D. N., Mongersun, A., Chau, H. & Araci, I. E. Tunable soft lithography molds enable rapid-prototyping of multi-height channels for microfluidic large-scale integration. *J. Micromechanics Microengineering.* **29**, 035009(2019).

[31] Lee YS, Bhattacharjee N, Folch A (2018). 3D-printed Quake-style microvalves and micropumps. Lab Chip.

[32] Glick CC (2016). Rapid assembly of multilayer microfluidic structures via 3D-printed transfer molding and bonding. Microsyst. Nanoeng..

[33] Compera N, Atwell S, Wirth J, Wolfrum B, Meier M (2021). Upscaling of pneumatic membrane valves for the integration of 3D cell cultures on chip. Lab Chip.

[34] Owens CE, Hart AJ (2018). High-precision modular microfluidics by micromilling of interlocking injection-molded blocks. Lab Chip.

[35] Razavi Bazaz S (2019). Rapid Softlithography Using 3D-Printed Molds. Adv. Mater. Technol..

[36] Venzac B (2021). PDMS Curing Inhibition on 3D-Printed Molds: Why? Also, How to Avoid It?. Anal. Chem..

[37] Guckenberger DJ, De Groot TE, Wan AMD, Beebe DJ, Young EWK (2015). Micromilling: A method for ultra-rapid prototyping of plastic microfluidic devices. Lab Chip.

[38] Jang M, Kwon YJ, Lee NY (2015). Non-photolithographic plastic-mold-based fabrication of cylindrical and multi-tiered poly(dimethylsiloxane) microchannels for biomimetic lab-on-a-chip applications. RSC Adv..

[39] Ogilvie, I. R. G. et al. Reduction of surface roughness for optical quality microfluidic devices in PMMA and COC. *J. Micromech. Microeng.* **20**, 065016 (2010).

[40] Johnson TJ, Ross D, Locascio LE (2002). Rapid microfluidic mixing. Anal. Chem..

[41] Mohammed M (2019). Studying the Response of Aortic Endothelial Cells under Pulsatile Flow Using a Compact Microfluidic System. Anal. Chem..

[42] Zheng C, Zhang X, Li C, Pang Y, Huang Y (2017). Microfluidic Device for Studying Controllable Hydrodynamic Flow Induced Cellular Responses. Anal. Chem..

[43] Bossink, E. G., Vollertsen, A. R., Segerink, L. I., Van Der Meer, A. D. & Odijk, M. Automated Medium Recirculation Using Macro Valves for High Flow Rates in an Endothelial Cell Culture Chip. in 1269–1270 (25th International Conference on Miniaturized Systems for Chemistry and Life Sciences, 2021).

[44] Festo. Technical manual, Solenoid valves MH1, miniature. https://www.festo.com/net/supportportal/files/10026/mh1.

[45] Goulpeau J, Trouchet D, Ajdari A, Tabeling P (2005). Experimental study and modeling of polydimethylsiloxane peristaltic micropumps. J. Appl. Phys..

[46] Li Y, Zhang D, Feng X, Xu Y, Liu BF (2012). A microsecond microfluidic mixer for characterizing fast biochemical reactions. Talanta.

[47] van der Helm, M. W., van der Meer, A. D., Eijkel, J. C. T., van den Berg, A. & Segerink, L. I. Microfluidic organ-on-chip technology for blood-brain barrier research. *Tissue Barriers* **4**, e1142493 (2016). 10.1080/21688370.2016.1142493 PMC4836466 27141422

[48] Sinha R (2016). Endothelial cell alignment as a result of anisotropic strain and flow induced shear stress combinations. Sci. Rep..

[49] Desai SY (2002). Mechanisms of endothelial survival under shear stress. Endothel. J. Endothel. Cell Res..

[50] Wong AD (2013). The blood-brain barrier: An engineering perspective. Front. Neuroeng..

[51] Dekker S (2018). Standardized and modular microfluidic platform for fast Lab on Chip system development. Sens. Actuators, B Chem..

