# Supplementary material for: Systematic characterization of cleanroom-free fabricated macrovalves, demonstrating pumps and mixers for automated fluid handling tuned for organ-on-chip applications
Source: Microsyst Nanoeng. 2022 May 23;8:54. doi: 10.1038/s41378-022-00378-y (PMC9124669; doi:10.1038/s41378-022-00378-y)
Supplement: Supplementary file 1 — Supplementary information [file 41378_2022_378_MOESM1_ESM.docx]

**Supplementary information**

**S.1 Troubleshooting**

The proposed fabrication method for our macrovalve is a straightforward and repeatable process. It is similar to the Quake-style valve, which is also used in several labs. However in practice, it appears that small, at first sight not important factors can influence the performance of the valve. We added this troubleshooting section to give possible explanations and guidance to help the researchers who want to fabricate the macrovalve themselves.

***Fig. S.1****: Cross section of the PDMS cast of the microfluidic flow layer without solvent treatment, smooth enough to be sealed. The staircase-like structure is still, but hardly, visible.*

**Problem 1 ‘Positive mold’:** The upstanding features in the mold for the flow layers are not rounded but a staircase-like structure with high steps is obtained. The steps of this structure are too high to be closed by the PDMS membrane, or higher than the 10 µm reported here in Fig. 2 or Fig. S.1 and S.3.

*Solution 1A:* This is mainly caused by the settings in HSMworks. In this paper, the step that mills the rounded structure is a 3D adaptive clearing step, using a 1 mm mill. Specifically, the fine step size (in the passes tab in HSMworks) determines the height of the staircase-like structure. We used for our molds the following settings in the passes tab: a tolerance of 0.1 µm, a step size of 0.1 mm, a fine step size of 0.01 mm and a smoothing tolerance of 0.01 µm. A cross section of the PDMS cast of the PMMA mold should look like Fig. S.1.

*Solution 1B:* If a smoother surface of the PMMA molds is required, a chloroform solvent treatment can be performed as described below, based on Ogilvie et al.^36^:

- Clean the PMMA mold first by wiping with a KimTech tissue with water, to remove the rough PMMA debris.
- Clean the PMMA mold by ultrasonication in DI water and detergent until all unwanted PMMA remains are removed.
- Rinse the PMMA mold with subsequent, clean DI water, ethanol and IPA.
- Dry the PMMS mold under a stream of nitrogen.
- Secure the cleaned PMMA mold to a glass Petri dish cover lid using Scotch tape.
- Pour chloroform into a Petri dish until the whole bottom surface of the dish is covered.
- Close the Petri dish with the cover lid with attached PMMA mold for 5 minutes.

Fig. 2 and Fig. S.3 show the smoothing effect of the solvent treatment on the surface of the PMMA mold.

*Solution 1C:* Treat the PMMA flow layer mold with a powder blasting at a very low pressure to remove burrs and loose unwanted PMMA, which could not be removed by the ultrasonic bath.

**Problem 2 ‘Valve’**: Valve does not close properly when applying a 1 – 1.5 bar pressure to the control channels.

*Solution 2:* The control layer is too thick, resulting in a thicker PDMS membrane at the site of the valve. As a result is the membrane not flexible enough to close the whole flow channel. The control layer can either be too thick due to a too low spinning speed and time, or to a too viscous PDMS. Possible causes of the PDMS becoming too viscous are when the 20:1 ratio is not exactly met, the degassing step was too long, or when PDMS is used which was stored in the fridge or freezer.

**Problem 3 ‘Valve’:** The valve breaks because the PDMS membrane/control layer ruptured after applying 1-1.5 bar to the control layer.

*Solution 3:* The control layer is too thin, resulting in a too thin PDMS membrane at the site of the valve. The cause of rupture of the PDMS membrane are opposite to the cause for a too thick PDMS membrane (Problem 2), and the solutions are also opposite to Solution 2. The PDMS is not viscous enough, which can be due to an incorrect 20:1 ratio or a degassing step, which was too short. Another possible cause is that the PMMA mold for the control layer is not cooled down to room temperature before spinning the PDMS. The higher temperature of the mold could make the PDMS less viscous and result in a thinner control layer. Also, please be aware that the spin-coating speeds depend on the height of the control channel and the size of the substrate^41^. Therefore, is in this article a different spinning speed used for the mixing and metering device compared to the devices with only a peristaltic pump or a single macrovalve and bridge.

***Fig. S.2****: Pumping rate of peristaltic pumps with a bit thicker membrane at different frequencies and pressures of actuation (6-phase pattern). 1.2 bar R^2^ = 0.9860, 1.5 bar R^2^ = 0.9998, 1.75 bar R^2^ = 0.9985*

**Problem 4 ‘Pump’:** The peristaltic pump does not reach the 45 µL/min at a frequency of 20 Hertz as reported in Fig. 4B, but a lower pumping rate, such as Fig. S.2, 1.2 bar.

*Solution 4:* The membrane between the control and flow layer is a bit too thick. The valve can still close, but it takes more time or pressure to fully seal the flow channel, resulting in a lower pumping capacity at a similar actuation pressure. A quick solution is to increase the actuation pressure. Causes and solutions for the too thick PDMS membrane are discussed before in Problem 2 and Solution 2.

Calibrating the pump is fairly easy, due to the linearity between the pumping rate and the actuation frequency per pressure. The calibration curves are stable over different measurements, as shown by the almost not visible error bars in Fig. 4B, 1 bar. Furthermore, when the pumping rate was examined for 20 or 45 minutes, similar pumping rate were observed. We advise to use the calibration method described in Fig. 8B, using the weight the outflow of the pump, over the use of flow sensors. Flow sensors have, relatively to the chip, a high hydraulic resistance, which will cause lower pumping rates measured.

**Problem 5 ‘Bond with glass slide’:** The control layer starts delaminating from the glass slide or the PDMS (1:10) layer after >1 hour actuation of the valves.

*Solution 5:* The bond between control layer and glass slide or the PDMS (1:10) layer is not good. After aligning the control and flow layer (step 6, Fig. 7), precure the two layers for 45 minutes at 60°C (instead of overnight). Meanwhile, spin-coat PDMS (1:10 (w/w)) on a glass slide at 300 rpm for 60 seconds and precure this layer for 20 minutes at 60°C. Remove the control and flow layer from the control layer PMMA mold and punch the inlets for the control channels. Place the control layer on the PDMB base layer without performing oxygen plasma treatment. After bonding, the chip can be cured in an oven at 60°C overnight.

**S.2 Dektak measurement**

The surface roughness of the rounded structure of the PMMA mold was examined by performing Dektak measurements. The measurement of the untreated structure is shown in Fig. S.3A, where, especially at the top of the rounded structure, clear vertical steps of 10 µm can be observed. A close-up of the top of the structure is shown in Fig. S.3B. The 10 µm steps correspond to the final step size as programed in HSMworks, explained in S.1 Solution 1A. Fig. S.3C shows the Dektak profile of the treated rounded structure, showing the smoothening effect of the chloroform solvent treatment, proposed as S.1 Solution 1B. The smoothened rounded structure in the mold provides a smoother rounded flow channel in the PDMS, which can allow better sealing of the macrovalve.

***Fig. S.3****: Dektak measurement of the PMMA mold with rounded protruding structure, directly micromilled, for the flow channel. Units of the graphs are vertically in nm and horizontally in µm.* ***A)*** *Untreated rounded structure in the PMMA mold. At the top of the structure (close-up shown in* ***B****), vertical steps of 10 µm are be observed, which correspond to the final step size programmed in HSMworks.* ***C)*** *The PMMA mold with rounded upstanding structure after treatment, showing the smoothening effect of the chloroform solvent treatment.*

**S.3 Systematic characterization of the flow channels**

*Tab. S.1: Cross sections of the PDMS casts of the flow channels.*

| **Flow channel width (µm)** | **Flow channel height (µm)** | | | | | | | |
| --- | --- | --- | --- | --- | --- | --- | --- | --- |
| **1000** | 50 | 100 | 200 | 300 | 400 | 500 | 600 | 700 |
|  |  |  |  |  |  |  |  |  |
| **750** | 37.5 | 75 | 150 | 225 | 300 | 375 | 450 | 525 |
|  |  |  |  |  |  |  |  |  |
| **500** | 25 | 50 | 100 | 150 | 200 | 250 | 300 | 350 |
|  |  |  |  |  |  |  |  |  |
| **250** | 12.5 | 25 | 50 | 75 | 100 | 125 | 150 | 175 |
|  |  |  |  |  |  |  |  |  |

Scale bars represent 500 µm for flow channel widths of 1000 µm and 750 µm. Scale bars represent 200 µm for flow channel widths of 500 µm and 250 µm.

*Tab. S.2: The actual measured dimensions (height and width) of the flow channel cross sections, with respect to the aimed design.*

| **Flow channel width (µm)** | |  | **Flow channel height (µm)** | | | | | | | | **Average** | **Standard deviation** |
| --- | --- | --- | --- | --- | --- | --- | --- | --- | --- | --- | --- | --- |
| **1000** | Height aim |  | 50 | 100 | 200 | 300 | 400 | 500 | 600 | 700 |  |  |
|  | Height measured |  | 50.533 | 100.970 | 185.588 | 280.969 | 392.906 | 477.299 | 582.224 | 691.624 |  |  |
|  | % difference |  | *1.07%* | *0.97%* | *-7.21%* | *-6.34%* | *-1.77%* | *-4.54%* | *-2.96%* | *-1.20%* | *2.75%* | *± 3.11%* |
|  |  |  |  |  |  |  |  |  |  |  |  |  |
|  | Width aim |  | 1000 | 1000 | 1000 | 1000 | 1000 | 1000 | 1000 | 1000 |  |  |
|  | Width measured |  | 1109.555 | 1093.492 | 1066.829 | 1055.909 | 1060.196 | 1057.167 | 1066.760 | 1067.970 |  |  |
|  | % difference |  | *10.96%* | *9.35%* | *6.68%* | *5.59%* | *6.02%* | *5.72%* | *6.68%* | *6.80%* | *7.22%* | *± 1.91%* |
| **750** | Height aim |  | 37.5 | 75 | 150 | 225 | 300 | 375 | 450 | 525 |  |  |
|  | Height measured |  | 37.315 | 72.468 | 151.519 | 213.096 | 294.204 | 357.660 | 444.325 | 518.040 |  |  |
|  | % difference |  | *-0.49%* | *-3.38%* | *1.01%* | *-5.29%* | *-1.93%* | *-4.62%* | *-1.26%* | *-1.33%* | *2.16%* | *± 2.13%* |
|  |  |  |  |  |  |  |  |  |  |  |  |  |
|  | Width aim |  | 750 | 750 | 750 | 750 | 750 | 750 | 750 | 750 |  |  |
|  | Width measured |  | 874.793 | 842.907 | 838.564 | 823.173 | 834.295 | 839.595 | 831.953 | 829.758 |  |  |
|  | % difference |  | *16.64%* | *12.39%* | *11.81%* | *9.76%* | *11.24%* | *11.95%* | *10.93%* | *10.63%* | *10.82%* | *± 2.08%* |
| **500** | Height aim |  | 25 | 50 | 100 | 150 | 200 | 250 | 300 | 350 |  |  |
|  | Height measured |  | 20.881 | 46.108 | 90.162 | 143.185 | 188.885 | 240.513 | 290.272 | 339.769 |  |  |
|  | % difference |  | *-16.48%* | *-7.78%* | *-9.48%* | *-4.54%* | *-5.56%* | *-3.79%* | *-3.24%* | *-2.92%* | *5.51%* | *± 4.59%* |
|  |  |  |  |  |  |  |  |  |  |  |  |  |
|  | Width aim |  | 500 | 500 | 500 | 500 | 500 | 500 | 500 | 500 |  |  |
|  | Width measured |  | 591.555 | 569.61 | 557.057 | 553.576 | 567.847 | 569.874 | 565.656 | 567.633 |  |  |
|  | % difference |  | *18.31%* | *13.92%* | *11.41%* | *10.72%* | *13.57%* | *13.97%* | *13.13%* | *13.53%* | *-12.31%* | *± 2.26%* |
| **250** | Height aim |  | 12.5 | 25 | 50 | 75 | 100 | 125 | 150 | 175 |  |  |
|  | Height measured |  | - | 24.245 | 40.622 | 54.790 | 82.342 | 107.578 | 133.936 | 167.975 |  |  |
|  | % difference |  | *-* | *-3.02%* | *-18.76%* | *-26.95%* | *-17.66%* | *-13.94%* | *-10.71%* | *-4.01%* | *-10.82%* | *± 8.50%* |
|  |  |  |  |  |  |  |  |  |  |  |  |  |
|  | Width aim |  | 250 | 250 | 250 | 250 | 250 | 250 | 250 | 250 |  |  |
|  | Width measured |  | - | 368.893 | 343.562 | 338.938 | 327.102 | 328.189 | 335.964 | 338.038 |  |  |
|  | % difference |  | *-* | *47.56%* | *37.42%* | *35.58%* | *30.84%* | *31.28%* | *34.39%* | *35.22%* | *32.23%* | *± 5.60%* |

Average off height	= -9.47 ± 6.77 µm

Average off width	= 79.89 ± 17.71 µm

<10% measured difference ; >10% measured difference

**S.4 Systematic characterization of the control channels**

*Tab. S.3: Cross sections of the PDMS casts of the control channels.*

| **For flow channel (µm)** | **Control channel width (µm)** | | | | |
| --- | --- | --- | --- | --- | --- |
|  | *Valve* | *Bridge* | *Valve* | *Bridge* | *Valve* |
| **1000**  ***(control channel height aim = 200)*** | 1000 | 100 | 600 | 120 | 400 |
|  |  |  |  |  |  |
| **750**  ***(control channel height aim = 150)*** | 750 | 75 | 450 | 90 | 300 |
|  |  |  |  |  |  |
| **500**  ***(control channel height aim = 100)*** | 500 | 50 | 300 | 60 | 200 |
|  |  |  |  |  |  |
| **250**  ***(control channel height aim = 50)*** | 250 | 25 | 150 | 30 | 100 |
|  |  |  |  |  |  |

Scale bars represent 500 µm for control channel widths of 1000 µm. Scale bars represent 200 µm for all other images.

*Tab. S.4: The actual measured dimensions (height and width) of the control channel cross sections, with respect to the aimed design.*

| **For flow channel width** | **Control channel width (µm)** |  | **Control channel width (µm)** | | | | | **Average** | **Standard deviation** |
| --- | --- | --- | --- | --- | --- | --- | --- | --- | --- |
| **1000** | Width aim |  | 1000 | 100 | 600 | 120 | 400 |  |  |
|  | Width measured |  | 1048.625 | 160.501 | 650.577 | 177.634 | 453.684 |  |  |
|  | % difference |  | *4.86%* | *60.50%* | *8.43%* | *48.03%* | *13.42%* | *27.05%* | *± 25.42%* |
|  |  |  |  |  |  |  |  |  |  |
|  | Height aim |  | 200 | 200 | 200 | 200 | 200 |  |  |
|  | Height measured |  | -37.359 | -37.303 | -28.786 | -36.258 | -34.135 |  |  |
|  | % difference |  | *-18.68%* | *-18.65%* | *-14.39%* | *-18.13%* | *-17.07%* | *-17.38%* | *± 1.79%* |
| **750** | Width aim |  | 750 | 75 | 450 | 90 | 300 |  |  |
|  | Width measured |  | 825.235 | 148.792 | 521.410 | 165.975 | 376.765 |  |  |
|  | % difference |  | *10.03%* | *98.39%* | *15.87%* | *84.42%* | *25.59%* | *46.86%* | *± 41.34%* |
|  |  |  |  |  |  |  |  |  |  |
|  | Height aim |  | 150 | 150 | 150 | 150 | 150 |  |  |
|  | Height measured |  | 111.409 | 114.511 | 116.753 | 114.616 | 118.848 |  |  |
|  | % difference |  | *-25.73%* | *-23.66%* | *-22.16%* | *-23.59%* | *-20.77%* | *-23.18%* | *± 1.85%* |
| **500** | Width aim |  | 500 | 50 | 300 | 600 | 200 |  |  |
|  | Width measured |  | 554.313 | 114.511 | 353.264 | 89.982 | 251.636 |  |  |
|  | % difference |  | *10.86%* | *129.02%* | *17.75%* | *49.97%* | *25.82%* | *46.69%* | *± 48.34%* |
|  |  |  |  |  |  |  |  |  |  |
|  | Height aim |  | 100 | 100 | 100 | 100 | 100 |  |  |
|  | Height measured |  | 85.627 | 82.397 | 85.627 | 84.591 | 78.176 |  |  |
|  | % difference |  | *-14.37%* | *-17.60%* | *-14.37%* | *-15.41%* | *-21.82%* | *-16.72%* | *± 3.15%* |
| **250** | Width aim |  | 250 | 25 | 150 | 30 | 100 |  |  |
|  | Width measured |  | 323.205 | 97.394 | 223.654 | 83.467 | 169.064 |  |  |
|  | % difference |  | *29.282%* | *289.576%* | *49.103%* | *178.223%* | *69.064%* | *-146.49%* | *± 111.00%* |
|  |  |  |  |  |  |  |  |  |  |
|  | Height aim |  | 50 | 50 | 50 | 50 | 50 |  |  |
|  | Height measured |  | 26.772 | 26.750 | 22.496 | 23.540 | 24.610 |  |  |
|  | % difference |  | *-46.46%* | *-46.50%* | *-55.01%* | *-52.92%* | *-50.78%* | *51.30%* | *± 3.64%* |

Average off width	= 61.68 ± 12.58 µm

Average off height	= -27.98 ± 8.33 µm

<10% measured difference ; >10% measured difference

***Fig. S.4****: Accuracy of the channels in the PDMS casts of the PMMA micromilled molds.* ***Left)*** *Summary of section S.3. The absolute error for the width of the flow channels is consistently approximately 80 µm, which can be easily corrected for in the micromilling process by adjusting the dimensions in the SolidWorks model. The absolute error of the height of the micromilled structures is between +5 µm and -25 µm, and its percentage difference is within a range of ±10%, except for the narrowest structures, mainly with a width of 250 µm.* ***Right)*** *Summary of section S.4. The absolute error of the control channel height is often approximately 25 µm - 30 µm. The control channel width is, similar as seen for the flow channels, too wide (<~80 µm).*

**S.5 Systematic characterization of the macrovalve actuation**

All four characterization chips have a range of control channel widths and flow channel heights (see Fig. 3A and B). By applying a ranging pressure to the control channels of all four chip versions, the valves and bridges were characterized (see Fig. S.5). This characterization shows us which dimensions should or could can be used to design PDMS chips with valves and/or bridges. The differences observed in the characterization between the chip versions, can be caused by the errors in the PMMA molds, introduced by micromilling (Fig. S.4). Also, the spinning speed of the PDMS for the control layer (Fabrication protocol Fig. 7 step 3b) can be tuned, which will result in a different characterization of the valve or chip. Fig. S.5A shows no data points for the narrowest flow channel height, as after micromilling almost no upstanding structure was left, resulting in the channel to directly collapse during fabrication.

***Fig. S.5****: Systematic characterization of all 4 chip versions.* ***A)*** *250 µm,* ***B)*** *500* *µm,* ***C)*** *750 µm,* ***D)*** *1000 µm. All data points have at least n = 3. Some data points were left out of consideration, because e.g. the control line was bonded to the glass layer and therefore the valve could not function.*

**S.6 Valve leakage**

The leakage of the valve was examined using a setup similar as shown in Fig. 8A, but with a flow sensor more precise in the low flowrate regime (Fluigent, FRP size S). To the flow channel, 20 mbar was applied, and to close the valve a pressure of 1.5 bar was applied to the control channel. The valve was opened, closed and opened for a time of 1 minute for each step, see Fig. S.6A. The average leakage when the valve is closed is 5.6 nL/min (n=4 measurements), below the detection limit of the flow sensor (10 nL/min). As the usual flow used in OoC applications is in the order of µL/min, a leakage in the order of nL/min, which is then approximately 0.1% of the flow in an ‘open valve’ situation, is adequate for closing purposes. Fig. S.6A+C also show the opening and closing behavior of a valve in time and thus the response time. A 90% response time of less than 0.5 seconds was measured. When closing, the flowrate drops in 1 second from 7.017 µL/min to 0.1133 µL/min; a drop of 98.4%.

The pressure applied to the flow channel also determines the (possible) valve leakage. To study this, the pressure to the flow channel was changed, while maintaining a constant pressure of 1.5 bar to the control channel, see Fig. S.6B. The pressure to the flow channel is divided into three regions. A pressure up to 150 mbar can be applied to the flow channel with no (significant) leakage (the green region). In the orange region of approximately 150 to 270 mbar, the valves may or may not leak. When applying 300 mbar, valve leakage of several µL/min was always observed.

***Fig. S.6****: Leakage of the macrovalve. A pressure of 1.5 bar was applied to the control channel and a pressure of 20 mbar was applied to the flow channel.* ***A)*** *Opening and closing behavior of a macrovalve with a chloroform treated flow layer.* ***B)*** *The measured flow rate in response to an increasing pressure applied to the flow channel, while maintaining the constant pressure on the control channel of 1.5 bar (n=3 measurements). A macrovalve with chloroform treated flow layer was used.* ***C)*** *Valve leakage of macrovalve with an untreated flow layer. With a closed valve, an average flow of 0.071 µL/min was measured.*

**S.7 Calibration curve mixing and metering device**

A relative background correction was performed, taking an area of the image, not at the site of the channel, as background area. The intensity of this background ($I_{{background}_{\#\%}}$) was considered for each picture and related to the background of the image with no food dye ($I_{{background}_{0\%}}$), providing a relative background correction factor:

$$Background correction factor (BCF)= \frac{I_{{background}_{0\%}}}{I_{{background}_{\#\%}}}$$

The measured intensity of the pixels in the region of interest were corrected with this background correction factor:

$$I= I_{measured} \cdot BCF$$

To establish a calibration curve, the corrected intensity was considered per pixel row in the region of interest, along the width of the channel for different concentrations of food dye, see Fig. S.7A. This intensity plot varies along the width of the channel (distance x), due to the rounded shape of the flow channel. Thereby a calibration curve was established per pixel row. Fig. S.7B-D show three calibration curves at various sites along the channel, all fitting a second order polynomial fit (R^2^ > 0.99). Using these calibration curves per pixel row, the concentration along the channel per pixel row can be calculated for unknown concentrations, as shown in Fig. 5. Incidentally, the I_0_/I was higher than the asymptote of the calibration curve, indicating that the concentration was higher than 100% in those pixel rows. In those cases, the concentration was set to be 100%.

***Fig. S.7:*** ***A)*** *Intensity along the width of the rounded flow channel per pixel row, as shown in Fig. 5. I_0_ is the intensity with 0% food dye, I the measured intensity at a specific concentration. The intensity along the width of the channel is not constant, due to the rounded shape of the channel. Therefore a calibration curve was obtained for each pixel row along distance x.* ***B+C+D)*** *The calibration curves of intensity I_0_/I against concentration food dye at specific sites along the channel, distance x. For each site x=10* ***(C)****, x=35* ***(D)****, and x=85* ***(B)****, a second order polynomial fit is observed, with a R^2^ of* ***(C)*** *0.9981,* ***(D)*** *0.9971 and* ***(B)*** *0.9994 respectively.*

**S.8 Video mixing**

 <https://youtu.be/WKpPdLS3lKY>

**S.9 Video dilution series**

 <https://youtu.be/13vEShHVHhI>

**S.10 Dilution factor**

To calculate the expected dilution factor, the volumes of the sections of the mixing loop were calculated and the ratio between the top and bottom side of the mixing loop was calculated to be 0.584:1. Fig. S.8 and Tab. S.5 provide the information to calculate this theoretical dilution factor. By adjusting the volume ratio in the chip, the dilution factor can be altered.

***Fig. S.8:*** ***A)*** *Schematic illustration of the volume ratio of top to bottom (0.584:1).* ***B)*** *Snapshot of the SolidWorks model of the mixing and metering flow layer to illustrate the sites of the rounded channel. The volume sections are indicated with numbers.*

*Tab. S.5: Calculation of volumes in mixing and metering device to determine the theoretical dilution factor.*

|  | *Top side* | *Bottom side* |
| --- | --- | --- |
| Number Fig. S.8 | (5+2+6) | (1+3+4) |
| Calculated volume | 2.37 mm^3^ | 4.06 mm^3^ |
| **Dilution factor** | **0.584** | **1** |

**S.11 Relevant parameters of the fabrication process**

*Tab. S.6: Relevant parameters of the fabrication process for different chips.*

|  | **Control channel height (µm)** | **Flow channel height (µm)** | **Flow channel width (µm)** | **Size chip (wxh) (mm)** | **Spinning speed (rpm)** |
| --- | --- | --- | --- | --- | --- |
| Test macrovalve + bridge | 200 | 400 | 1000 | 15x15 | 500 |
| Peristaltic pump | 200 | 400 | 1000 | 15x15 | 500 |
| Mixing and metering device | 200 | 400 | 1000 | 15x30 | 400 |
| Characterization chip 1000 | 200 | Varying | 100 | 30x90 | 300 |
| Characterization chip 750 | 150 | Varying | 750 | 30x90 | 400 |
| Characterization chip 500 | 100 | Varying | 500 | 30x90 | 600 |
| Characterization chip 250 | 50 | Varying | 250 | 30x90 | 1000 |
| Recirculation chip | 200 | 200 | 1000 | 15x30 | 400 |
